# Polo-Like Kinase 1 Inhibits the Activity of Positive Transcription Elongation Factor of RNA Pol II b (P-TEFb)

**DOI:** 10.1371/journal.pone.0072289

**Published:** 2013-08-16

**Authors:** Liangzhen Jiang, Yan Huang, Min Deng, Ting Liu, Wenbin Lai, Xin Ye

**Affiliations:** 1 Center for Molecular Immunology, CAS Key Laboratory of Pathogenic Microbiology and Immunology, Institute of Microbiology, Chinese Academy of Sciences, Beijing, P. R. China; 2 Graduate University of Chinese Academy of Sciences, Beijing, P. R. China; Florida State University, United States of America

## Abstract

Polo-like kinase 1 (Plk1) is a highly conserved Ser/Thr kinase in eukaryotes and plays a critical role in various aspects of the cell cycle. Plk1 exerts its multiple functions by phosphorylating its substrates. In this study, we found that Plk1 can interact with cyclin T1/Cdk9 complex-the main form of the positive transcription elongation complex b (P-TEFb), and its C-terminal polo-box domain is responsible for the binding. Further analysis indicated that Plk1 could phosphorylate cyclin T1 at Ser564 and inhibit the kinase activity of cyclin T1/Cdk9 complex on phosphorylation of the C-terminal domain (CTD) of RNA polymerase II. By taking the approach of luciferase assay, we demonstrated that over-expression of both wild type Plk1 and constitutively active form of Plk1 inhibits the P-TEFb dependent HIV-1 LTR transcription, while knockdown of Plk1 increases the HIV-1 LTR transcription. Consistently, the data from the HIV-1 pseudovirus reporter assay indicated that Plk1 blocks the gene expression of HIV-1 pseudovirus. Taken together, our results revealed that Plk1 negatively regulates the RNA polymerase II-dependent transcription through inhibiting the activity of cyclin T1/Cdk9 complex.

## Introduction

Cell cycle progression is precisely regulated and requires the coordination of multiple events tightly controlled by protein kinases including polo-like kinase 1 (Plk1). Plk1 belongs to a highly conserved family of Ser/Thr kinases and plays an essential role in various aspects in mitosis, such as mitotic entry, spindle pole functions, chromosome segregation and cytokinesis [1,2]. Plk1 has emerged as a novel player beyond mitosis in maintaining genomic stability during DNA replication and as an important modulator of the DNA damage checkpoint [3].

Eukaryotic gene transcription is significantly silenced through all of the three nuclear RNA polymerases when cells enter into mitosis [4]. Several hypotheses have been put forward to explain the molecular repression mechanisms [5]: inhibitory phosphorylation of basal transcription factors and/or RNA polymerases in mitosis to prevent various aspects of transcription: initiation, elongation or termination plays a major role [6–8]. CDK1/cyclin B1, the essential mitotic kinase is believed to be the master kinase to silence transcription in mitosis. For instance, phosphorylation of Cdk7 in the T-loop by Cdk1/cyclin B1 will cause the inhibition of the TFIIH-associated kinase and transcription activities [9]. These data suggested that the phosphorylation of transcription apparatus functions as a direct link between the regulation of transcription and the cell cycle.

RNA Pol II-dependent transcription elongation is positively regulated by the positive transcription elongation factor b (P-TEFb) [10]. P-TEFb stimulates transition from abortive to productive transcription elongation by preferentially phosphorylating Ser2 of the 52 heptapeptide repeats (YSPTSPS) of the C-terminal domain (CTD) of the largest subunit of RNA Pol II to promote transcription [10]. In addition, P-TEFb phosphorylates the negative transcription elongation factors DSIF and NELF to release their blocking [11,12]. P-TEFb is a heterodimer primarily comprised of Cdk9 and cyclin T1, or cyclin T2 and cyclin K in some cases [13]. P-TEFb kinase activity has also been linked to specific events such as human immunodeficiency virus type 1 (HIV-1) and T-Lymphotropic Virus Type 1(HTLV-1) replication [14,15], and cardiac hypertrophy [16]. In the case of transcription of HIV-1, P-TEFb is recruited to RNA Pol II through binding of cyclin T1 with Tat and the bulge-loop within TAR (transactivation response element) sequence and is required for the transcription of viral genes [17]. P-TEFb exists in two forms, the active cyclin T1/Cdk9 heterodimer and an inactive 7SK snRNP in which cyclin T1/Cdk9 activity is sequestered by complexing with the 7SK small nuclear RNA(snRNA) and HEXIM1 [18,19]. The active form of P-TEFb is recruited to gene promoters through Brd4. Brd4 is a bromodomain protein which is capable of binding acetylated histones and is implicated in the transmitting the epigenetic memory through mitosis [20]. Brd4 recruits P-TEFb by contacting acetylated chromatin and the Mediator complex, and enhances P-TEFb-dependent phosphorylation of the RNA Pol II CTD and transcriptional activation [21,22].

As Plk1 is strongly linked with mitotic progression, we sort to identify the relevance between Plk1 and the RNA Pol II-dependent transcription apparatus. In this study, we demonstrated that Plk1 can associate with P-TEFb complex and phosphorylate cyclin T1. We provided evidences to show that Plk1 suppresses P-TEFb kinase activity towards CTD of RNA Pol II and inhibits RNA Pol II-dependent transcription. Our results suggest that Plk1 functions as a negative regulator on transcription through phosphorylating cyclin T1.

## Materials and Methods

### Plasmids and Antibodies

pCMV FLAG-Plk1 and its mutants and bacteria expression plasmids pET-30a-Plk1, pET-30a-Plk1 TD (constitutively active form of Plk1) and pET-30a-Plk1 KD (kinase deficient form of Plk1) were generated as described previously [23]. pCMV myc-Plk1 were made by cloning Plk1 cDNA into the pCMV myc vector (BD Clontech) at EcoRI-XhoI sites. pCMV FLAG-Cdk9, Cdk7, and cyclin T1 were constructed by cloning the cDNA by PCR from human embryo kidney cDNA library into the pFLAG-CMV2 vector (Sigma) at EcoRI-XhoI, EcoRI-KpnI and EcoRI-BamHI sites respectively. To generate expression plasmids in bacteria, the deletion(1-240, 241-480, 481-630, 631-726, 361-505, 480-600, 480-530, and 531-630) and point mutants of cyclin T1(S564A and S564D) were generated by PCR from full-length cyclin T1 cDNA and cloned into pET-41c (Novagen) at EcoRI-XhoI sites. GST-fused Cdk9 expression plasmids were generated by PCR from full-length cDNA and inserted into pGEX-6p-1 at EcoRI-XhoI sites. Plk1 polo-box domain (PBD) (330–603) and PBD H538A/K540A mutant were cloned into pET-41b at EcoRI-XhoI sites. pGST-RNA Pol II CTD is a kind gift from Prof. Peterlin B.M. [24]. The HIV-1 long terminal repeat (LTR)-based luciferase reporter plasmid G5-83-HIV-luc was kindly provided by Prof. Wong J [25]. FLAG antibody (M2) was purchased from Sigma. Myc (9E10), Plk1 (F-8), β-actin (1–19), Cdk9(C-20), cyclin T1(T-18) and phospho-Ser histone H3 (sc-8656-R) antibodies were purchased from Santa Cruz Biotechnology. Anti-phosphoserine antibody (AB1603) was purchased from Millipore.

### Cell Culture and Synchronization

293T, HeLa, HCT116 and NIH3T3 cells were obtained from American Tissue Culture Collection (ATCC) and maintained in Dulbecco’s modified Eagle’s medium (DMEM) supplemented with 10% fetal bovine serum (PAA) and 100 µg/ml streptomycin and 100 U/ml penicillin at 37 °C with 5% CO_2_. HCT116 cells were synchronized at G1/S phase by treatment with double thymidine in the same way as for U2OS cells described in the previous report [26]. Briefly, cells were synchronized at G1/S transition by treatment with 2 mM thymidine for 16 h in complete medium, released in fresh medium for 8 h and then incubated with 2 mM thymidine for another 16 h. Cells were synchronized to S phase after released for 3h from double thymidine treatment. To obtain cells synchronized in mitosis, cells were incubated for 16 h in the presence of 2 mM thymidine and released for 6 h followed by treatment with 100 ng/ml Nocodazole for 6h.

### Co-immunoprecipitation

293T cells were transfected with indicated plasmids for 24 h and lysed in lysis buffer (50 mM TrisCl, pH 8.0, 150 mM NaCl, 0.5% Triton X-100) with protease inhibitor (Roche Applied Science) at 4 °C for 15 min. Then the cell lysates were incubated with Plk1 antibody at 4 °C for 2 h followed by addition of protein A-agarose beads for 1h or with FLAG antibody (M2, Sigma) or Myc antibody (Genomics Technology) conjugated agarose beads at 4°C for 4 h. The immunoprecipitates were subjected to immunoblotting with indicated antibodies.

### Protein Purification and GST Pull-down Assays

GST-Cdk9, GST-Plk1-PBD, GST-Plk1-PBD H538A/K540A, GST-RNA Pol II CTD (GST-Pol II CTD), GST-cyclin T1 and its mutants were purified from *Escherichia coli* (BL21) and immobilized on the Sepharose 4B-glutathione beads (Pharmacia). His-tagged Plk1, Plk1 TD or Plk1 KD were purified from *Escherichia coli* (BL21) with nickel-nitrilotriacetic acid beads (Qiagen) and eluted with 300 nM imidazole (pH 7.4). The eluted protein was dialyzed with dialysis buffer (50mM TrisCl, pH7.5, 100 mM NaCl). For the GST pull-down assay, equal amount of immobilized GST-tagged protein or GST was incubated with His-Plk1 or cell lysate at 4°C for 1 h followed by washing with lysis buffer. The bound proteins were subjected to immunoblotting with indicated antibody.

### In Vitro Kinase Assay

Purified GST-cyclin T1 or GST-cyclin T1 mutants were incubated with His-Plk1 TD or His-Plk1 KD in kinase buffer (50 mM TrisCl, pH 7.5, 10 mM MgCl2, 1 mM DTT) with 1 mM cold ATP, 1 mCi of [γ-^32^P] ATP at 30 °C for 30 min, and then washed with kinase buffer. The beads were resolved by 10% SDS-PAGE and subjected to autoradiography. For cyclin T1/Cdk9 *in vitro* kinase assay, FLAG-Cdk9 and FLAG-cyclinT1 were immunoprecipated from pCMV FLAG-cyclin T1 and pCMV FLAG-Cdk9 transfected 293T cells, and incubated with purified GST-Pol II CTD as substrates, in the presence or absence of purified His-Plk1 TD or His-Plk1 KD in kinase buffer at 30°C for 30 min. The samples were resolved by 8% SDS-PAGE and subjected to autoradiography.

### Mass Spectrometry

GST-cyclin T1 was incubated with His-Plk1 TD for *in vitro* kinase assay as described above in the presence of cold ATP. Subsequently, GST-cyclin T1 was isolated using SDS-PAGE and then trypsinized. The tryptic peptides were analyzed by HPLC-ESI/MS/MS with a Thermo Finnigan LTQ adapted for nanospray ionization. The tandem spectra were searched against NCBI Human database reference database(2011/12/14) using the SEQUEST (BioworksBrowser 3.3.1 SP1). SEQUEST was searched with peptide tolerance of 3 Amu and fragmentation tolerance of 1 Amu. Results was ﬁltered by Xcorr +1 >1.5, +2 > 2.0, + 3>2.5, preliminary score(Sp) > 500, Delta Cn < 0.1, Rsp > 5. The phosphpeptides was checked manually.

### Flow Cytometry

Cells were harvested, fixed with ice-cold 70% ethanol at -20°C, and stained with PBS/1% BSA containing 20 mg/ml of propidium iodide and 10 mg/ml of RNase A. Stained cells were analyzed on a FACS instrument (BD FACS Calibur).

### RNA Interference

293T cells were transfected with Plk1-specific small interfering RNA (siRNA) or control siRNA (30 nM) (Ribobio) for 48 h using Lipofectamine 2000 (Invitrogen). Three Plk1 siRNA were synthesized corresponding to the following cDNA sequences: 5’-CAACCAAAGTCG AATATGA-3’ (Plk1 siRNA-1); 5’-CCTCACAGTCCTCAATAAA-3’ (Plk1 siRNA-2); 5’-CCTTAAATATTTCCGCAAT-3’ (Plk1 siRNA-3).

### Luciferase Assay

293T cells in a 24-well plate were transfected with plasmids for expressing Plk1, Plk1 TD or Plk1 KD and HIV-1 luciferase reporter G5-83-HIV-luc for 48 h. The cells were harvested and divided into 2 sets evenly. One set was subjected to luciferase assay according to the manufacturer’s instructions using firefly luciferase kit (E4030, Promega). The DNA from the other set was extracted and subjected to real-time PCR to quantify the luciferase DNA with GAPDH as the internal control. The luciferase activity was measured and normalized to the amount of luciferase DNA. The primers used for the real-time PCR are: GAPDH, sense 5’ –TGCACCACCAACTGCTTAG-3’; antisense 5’-GATGCAGGGATGATGTTC-3’, and luciferase, sense 5’-AGAGATACGCCCTGGTTCC-3’; antisense 5’-GATGCAGGGATGATGTTC-3’.

### Pseudovirus Infection

To prepare vesicular stomatitis virus G protein (VSV-G)-pseudotyped HIV-1 luciferase reporter virus, 293T cells were transfected with the HIV-1 core plasmid pNL4.3-Luc (E^-^R^-^) and VSV-G envelope protein expression plamids. 48 h post-transfection, the supernatants were collected and used for infection. For virus infection, the HCT116 cells were plated in a 24-well plate (8 x10^5^ cells/well) and infected with VSV-G-pseudotyped HIV-1 reporter virus at 37 °C for 1 h. Then the cells were washed with PBS and cultured in fresh DMEM/10% FBS for 48 h. The cells lysates were harvested and subjected to luciferase assay.

## Results

### Plk1 interacts with P-TEFb

**Figure 1:**
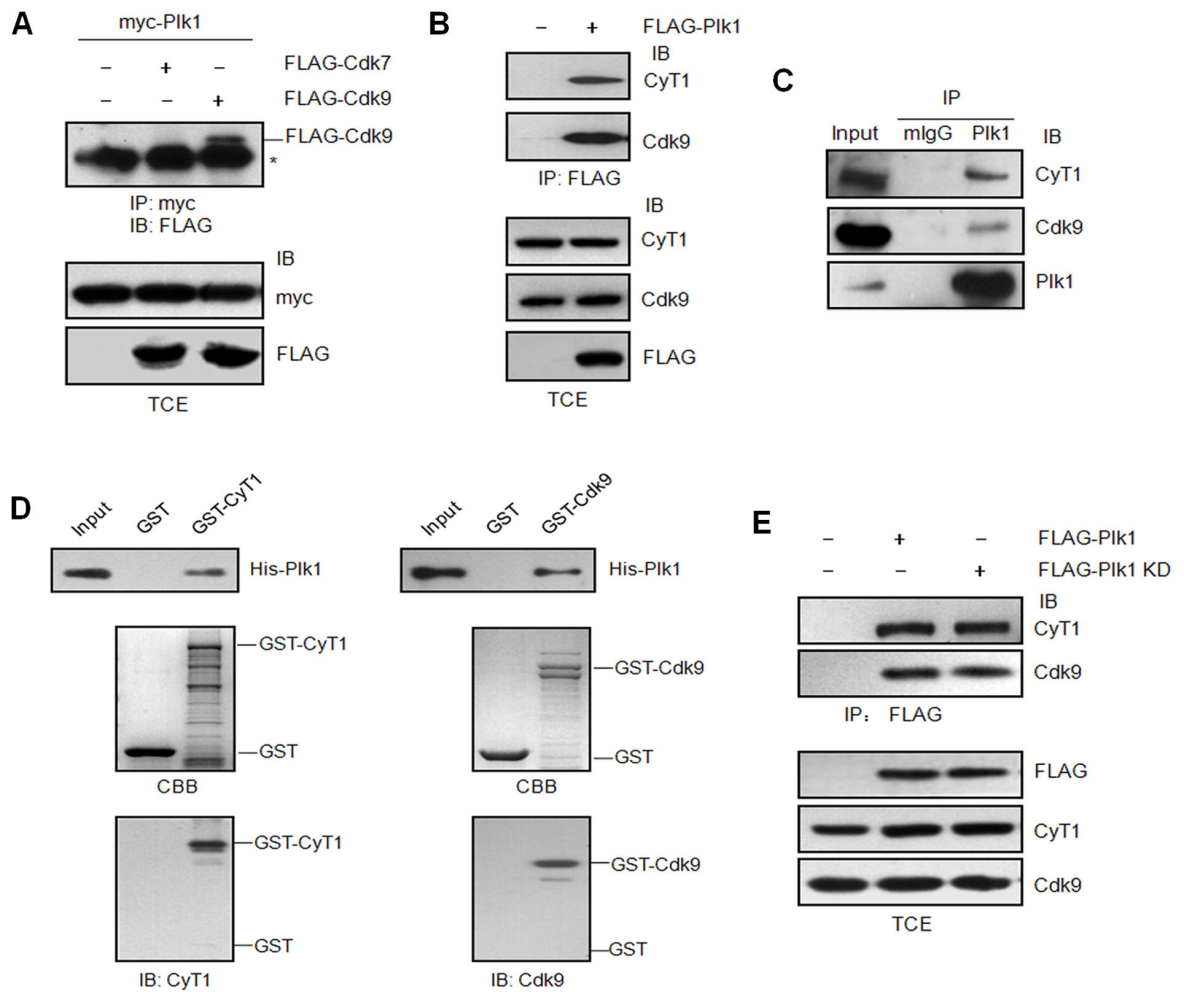
Plk1 interacts with P-TEFb independent of it kinase activity. (A) 293T cells were transfected with pCMV myc-Plk1 and pCMV FLAG-Cdk7 or FLAG-Cdk9, or empty vector as the control. The total cell extracts (TCE) were immunoprecipitated (IP) with myc antibody and immunoblotted (IB) with FLAG antibody. Asterisk indicates cross-reacting unrelated band. (B) 293T cells were transfected with empty vector or pCMV FLAG-Plk1. The total cell extracts were immunoprecipitated with FLAG antibody and immunoblotted with Cdk9 and cyclin T1 antibodies. The empty vector was used as the negative control. (C) HeLa cells were synchronized in M phase by Nocodazole treatment. The cell lysates were harvested and immunoprecipitated with normal mouse serum or Plk1 antibody, and then immunoblotted with Cdk9, cyclin T1 and Plk1 antibodies respectively. (D) GST pull-down assay. Purified His-Plk1 was incubated with immobilized GST, GST-cyclin T1 or GST-Cdk9 respectively. The bound protein was detected by immunoblotting with Plk1 antibody. *CBB*, Coomassie Brilliant Blue. (E) 293T cells were transfected with pCMV FLAG-Plk1, pCMV FLAG-Plk1 KD (K82R) or empty vector as the control. The cell lysates were immunoprecipitated with FLAG antibody and immunoblotted with Cdk9 and cyclin T1 antibodies.

It is known that RNA Pol II-dependent transcription is silenced in mitosis [27], while as an essential mitotic kinase, Plk1 activity peaks during G2/M phase [1]. We wonder if there is any relevance between Plk1 and the RNA Pol II transcription apparatus. There are two kinase complexes which play critical roles in RNA Pol II-dependent transcription-cyclin H/Cdk7 and P-TEFb (cyclin T1/Cdk9). Therefore we first examined whether Plk1 interacts with either of them by co-immunoprecipitation assay. The data showed Plk1 could bind the elongation complex (P-TEFb), but not the initiation CDK complex (cyclin H/Cdk7) (Figure 1A, B). To further confirm the interaction between endogenous Plk1 and P-TEFb, HeLa cells were synchronized by Nocodazole in M phase. Then the cell lysates were immunoprecipitated with Plk1 antibody followed by immunoblotting with Cdk9, cyclin T1 or Plk1 antibodies. The results further verified that Plk1 could interact with P-TEFb complex (Figure 1C). To examine if the interaction between Plk1 and P-TEFb complex is direct or not, we took the approach of GST pull-down assay with GST-Cdk9, GST-cyclin T1 and His-tagged Plk1. The data indicated that both Cdk9 and cyclin T1 could interact with Plk1 directly *in vitro* (Figure 1D). To identify if the kinase activity of Plk1 is involved in the interaction, FLAG-tagged Plk1 and the kinase-defective mutant FLAG-tagged Plk1 KD were expressed in 293T cells respectively and the cell lysates were immunoprecipitated with FLAG antibody followed by immunoblotting with Cdk9 and cyclin T1 antibodies. As shown in Figure 1E, the interaction between Plk1 and P-TEFb complex is independent of Plk1 kinase activity.

**Figure 2:**
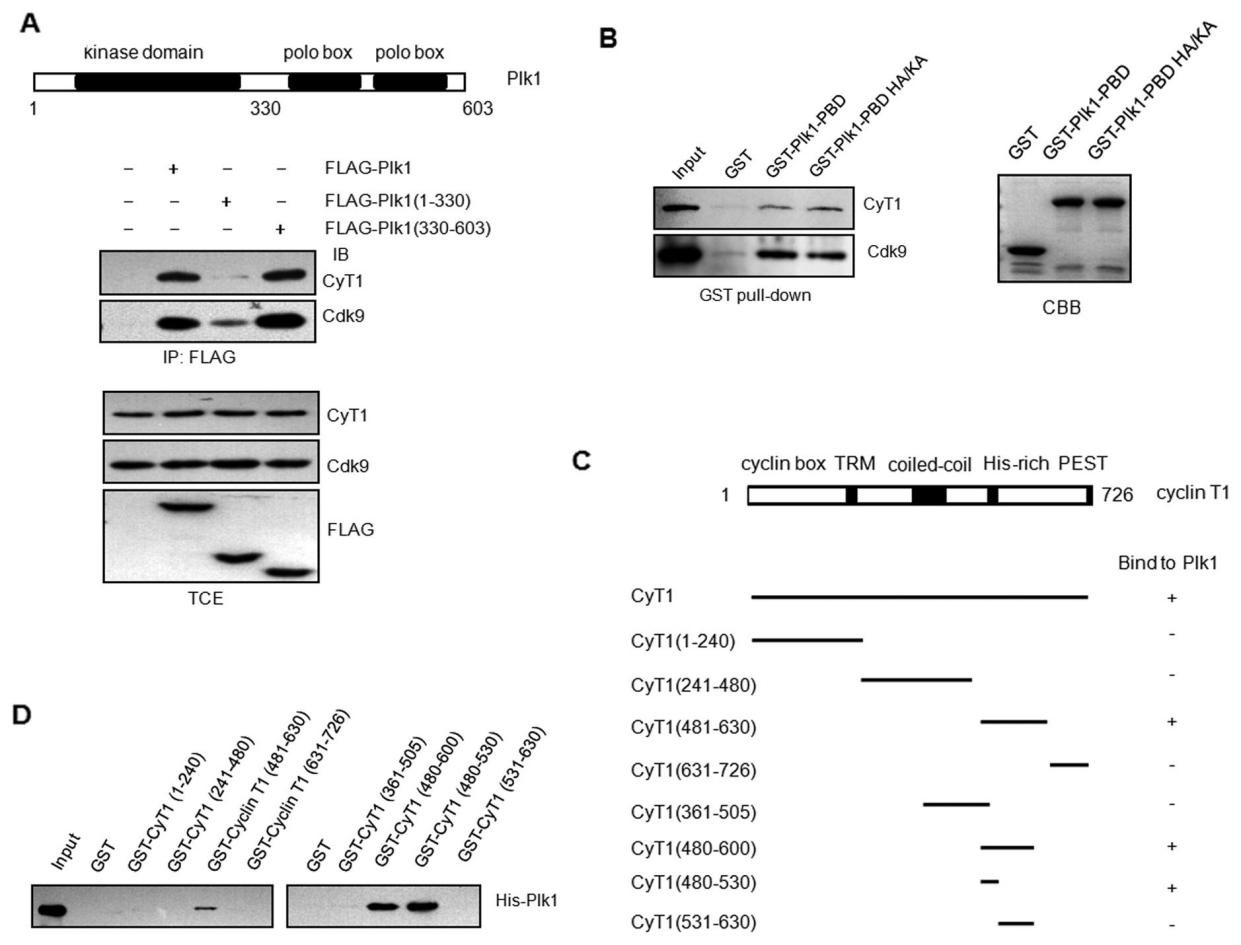
Identification of binding region of Plk1 and cyclin T1. (A) 293T cells were transfected with pCMV FLAG-Plk1or the indicated Plk1 deletion mutants, or empty vector as the control. The total cell extracts were immunoprecipitated with FLAG antibody and immunoblotted with Cdk9 and cyclin T1 antibody respectively. (B) GST pull-down assay. Equivalent amount of cell lysates from 293T cells were incubated with immobilized GST, GST-Plk1 polo-box domain (PBD) or GST-Plk1 PBD H538A/K540A, whose residues crucial for PBD phosphopeptide binding were mutated to alanine, followed by immunoblotting with Cdk9 and cyclin T1 antibody. (C) Structure of human cyclin T1 and cyclin T1 truncations. (D) GST-cyclin T1 and its truncations and His-Plk1 were purified and subjected to GST pull-down assay.

Plk1 consists of N-terminal kinase domain and two polo-box domains at the C terminus. To determine which region of Plk1 is required for its interaction with P-TEFb, pCMV FLAG-Plk1(1-330) and pCMV FLAG-Plk1(330-603) were generated as described previously [23] (Figure 2A) and transfected into 293T cells. The data from immunoprecipitation demonstrated that the C-terminal polo-box domains of Plk1 are mainly responsible for its binding with P-TEFb complex, while its N-terminal region shows very weak interaction with P-TEFb. To analyze if the prime phosphorylation of P-TEFb is required for the interaction between Plk1 and P-TEFb, the GST-Plk1 PBD and GST-Plk1 PBD H538A/K540A in which two residues crucial for Plk1 binding to phosphopeptide [28] were mutated to alanine were generated. It seems that prime phosphorylation of P-TEFb is not required for its interaction with Plk1 since GST-Plk1 PBD H538A/K540A mutant exhibits the same binding intensity with P-TEFb as wild-type Plk1 PBD (Figure 2B). The above data indicate that Plk1 can bind with P-TEFb without prime phosphorylation.

Human cyclin T1 contains a cyclin box at the N terminus, a coiled-coil sequence, a histidine-rich region and a PEST sequence at the C terminus (Figure 2C). The cyclin box is essential for its binding with Cdk9. The cyclin T1(251-272) named Tat recognition motif (TRM) is essential for its binding with HIV-1 Tat and TAR [29], which is critical for HIV-1 transcription. Histidine-rich region of cyclin T1 is responsible for its interaction with RNA Pol II CTD [24]. Proteins that bind cyclin T1 in the histidine region play regulatory roles in P-TEFb activity [30,31].

To address which region of cyclin T1 is required for its association with Plk1, four of cyclin T1 truncated mutants were generated for GST pull-down assay. The data demonstrated that cyclin T1 (481–630) is critical for its binding with Plk1 (Figure 2D). GST pull-down assay with shorter forms of cyclin T1 further indicated that cyclin T1(506-530) which is exactly the histidine-rich region is sufficient for its interaction with Plk1(Figure 2D).

### Cyclin T1 Is Phosphorylated by Plk1

**Figure 3:**
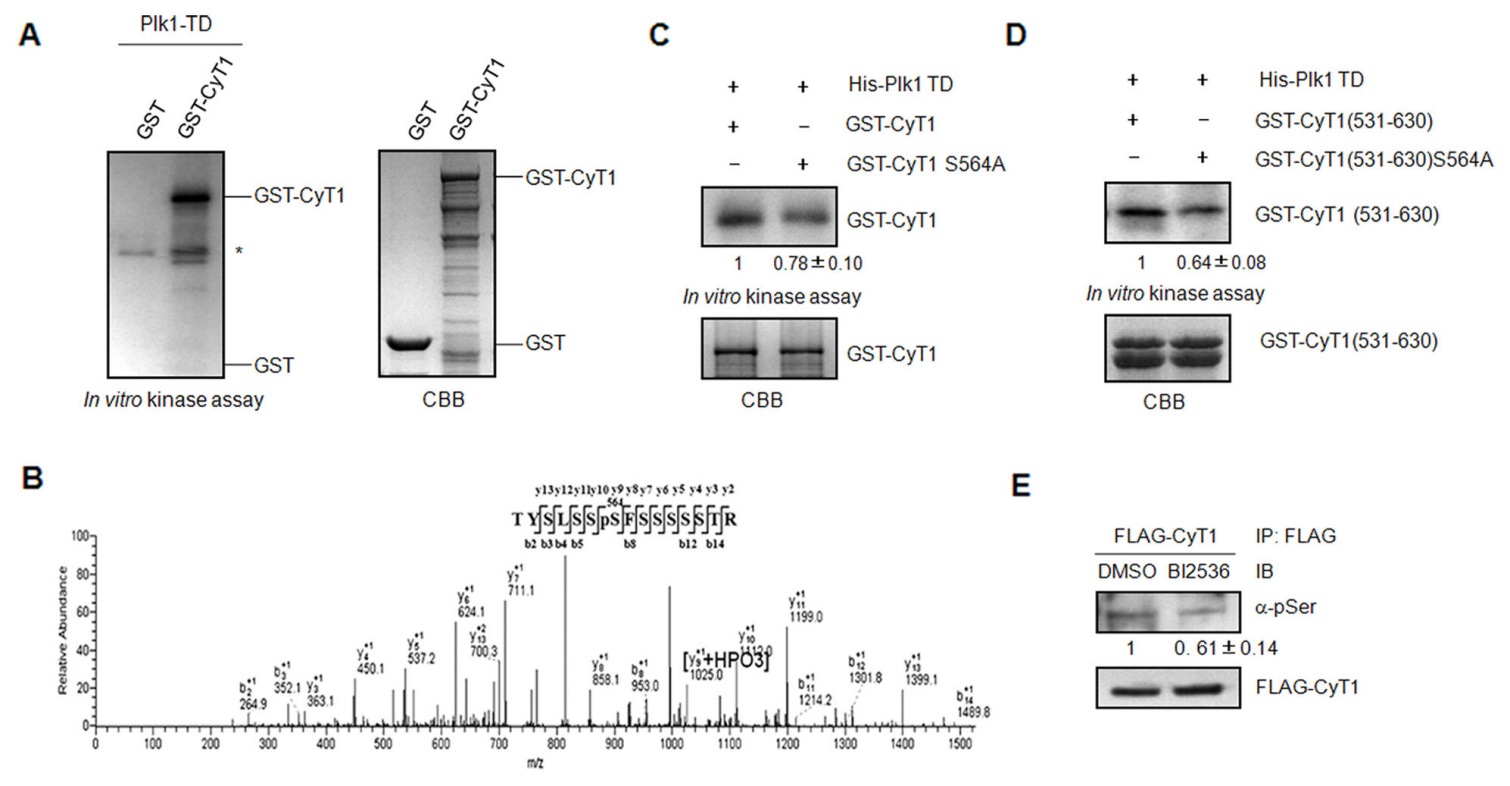
Plk1 phosphorylates cyclin T1 at Ser564. (A) Purified GST or GST-cyclin T1 were incubated with His-Plk1 TD for the *in vitro* kinase assay. *CBB*, Coomassie Brilliant Blue. Asterisk indicates Plk1 autophosphorylation. (B) Identification of phosphorylation site(s) of cyclin T1 by HPLC-ESI/MS/MS spectrometry. Purified GST-cyclin T1 was incubated with His-Plk1 TD for the *in vitro* kinase assay in the presence of cold ATP and then subjected to mass spectrometry analysis. [y_9_+HPO_3_] indicates phosphorylation with an increasement of 80 mass unit. (C, D) Purified GST-cyclin T1 and GST-cyclin T1 S564A mutant (C) or GST-cyclin T1(531-630) and GST-cyclin T1(531-630) S564A (D) were incubated with His-Plk1 TD in the presence of [γ-^32^P] ATP for the *in vitro* kinase assay. *CBB*, Coomassie Brilliant Blue staining. The phospho-signal was normalized to the total amount of GST-cyclin T1 or its mutants. (E) 293T cells were transfected with pCMV FLAG-cyclin T1 for 36 h and then treated with DMSO or BI2536 (1µM) for 3.5 h before harvest. Cell lysates were immunoprecipitated with FLAG antibody and followed by immunoblotting with anti-phospho-Serine and FLAG antibodies. The phospho-signal was normalized to the total amount of FLAG-cyclin T1. The quantification is represented as the mean ± SD from three independent experiments. Statistical significance was determined by Students t-test (*p value* < 0.05).

It has been reported that both Cdk9 and cyclin T1 can be phosphorylated and their phosphorylation is related to their function [15,32–34]. To examine if Cdk9 and/or cyclin T1 are the substrates of Plk1, bacteria-expressed GST-Cdk9, GST-cyclin T1 and constitutive active form of Plk1(His-Plk1 TD) were prepared for *in vitro* kinase assay. The results showed that cyclin T1, but not Cdk9 can be phosphorylated by Plk1 *in vitro* (Figure 3A and data not shown). In many cases, substrates of Plk1 need to be prime phosphorylated by other kinases to create a binding platform for Plk1 and the subsequent phosphorylation [35], while some substrates of Plk1 do not need the prime phosphorylation such as YY1 [36]. It seems that cyclin T1 phosphorylation by Plk1 does not need to be prime phosphorylated, which is consistent with the data shown in Figure 2B that the interaction between cyclin T1 and Plk1 does not need the prime phosphorylation of cyclin T1. To determine the phosphorylation site of cyclin T1 by Plk1, we took the approach of mass spectrometry analysis on purified GST-cyclin T1 after subjecting it to *in vitro* kinase assay with His-Plk1 TD. The data indicated that Ser564 at cyclin T1 is phosphorylated by Plk1 (Figure 3B). To further verify the phosphorylation site, we generated full length GST-cyclin T1 S564A mutant and shorter form of GST-cyclin T1 (GST-cyclin T1(531-630)) and its mutant (GST-cyclinT1(531-630) S564A) and performed *in vitro* kinase assay with His-Plk1 TD. As shown in Figure 3C and 3D, there is a significant reduction of phosphorylation on cyclin T1 S564A mutants compared to that on wild type of cyclin T1. However, cyclin T1(S564A) can still be phosphorylated by Plk1, suggesting that there could be other phosphorylation site(s) on cyclin T1 by Plk1 which are not identified by the approaches applied.

In order to examine if cyclin T1 is phosphorylated by Plk1 *in vivo*, 293T cells transfected with FLAG-cyclin T1 were treated with or without Plk1 inhibitor BI2536. To avoid the influence of cell cycle profile change, we treated the cells with BI2536 for a short time (3.5h). The flow cytometry data showed that BI2536 treatment causes a slight increase of cells in G2/M phase (data not show). The cell lysates were immunoprecipated with FLAG antibody and immunoblotted with phospho-serine antibody. The data showed that the phosphorylation of cyclin T1 on serine is reduced in BI2536 treated cells (Figure 3E).

### Phosphorylation of cyclin T1 by Plk1 inhibits the P-TEFb kinase activity

**Figure 4:**
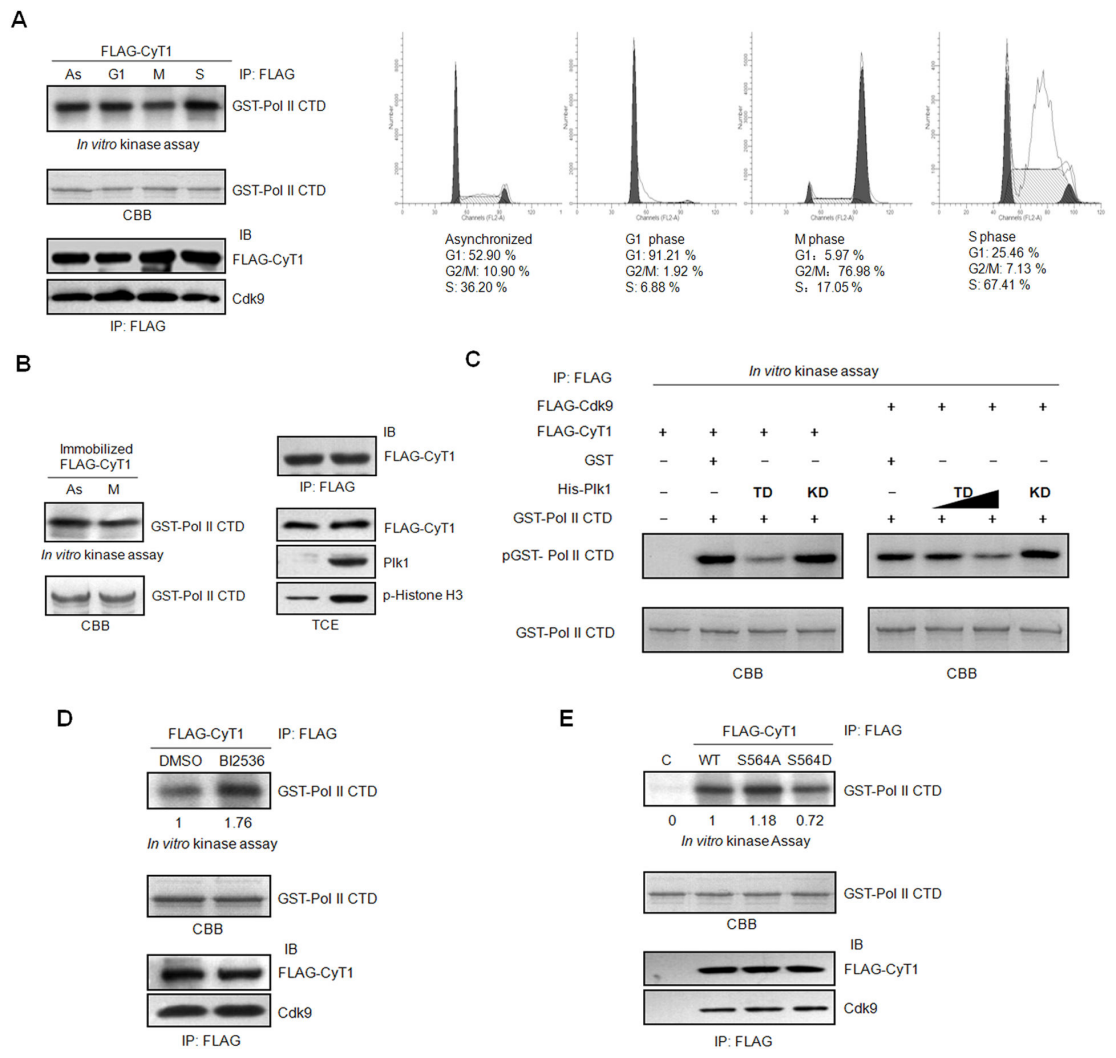
Plk1 inhibites the kinase activity of the P-TEFb complex. (A) *In vitro* kinase assay. HCT116 cells transfected with pCMV FLAG-cyclin T1 were synchronized into different phases as described in Material and Method. The synchronization of the cells was detected by FACS. Cell lysates were immunoprecipated with FLAG antibody. Half of the immunoprecipates were subjected to immunoblotting with Cdk9 and FLAG antibody. The other half of the immunoprecipates were then incubated with GST-RNA Pol II CTD in the presence of [γ-^32^P] ATP for the *in vitro* kinase assay. (B) FLAG-cyclin T1 over-expressed in 293T cells were immunoprecipated with FLAG antibody. The immunoprecipitated cyclin T1 complexes were preincubated with the lysates from HeLa cells either asynchronized or synchronized in M phase with cold ATP for the *in vitro* kinase assay, and washed with kinase buffer. Then the FLAG-cyclin T1 complexes were incubated with GST-RNA Pol II CTD and [γ-^32^P] ATP for a second round of *in vitro* kinase assay. The expression level of Plk1, phosphorylation of histone H3 in HeLa cells and the immunoprecipitated FLAG-cyclin T1 after incubating with Hela cell extracts and washed were detected by immunoblotting with the indicated antibody. (C) 293T cells were transfected with pCMV FLAG-cyclin T1 or pCMV FLAG-Cdk9 respectively. The cell lysates were immunoprecipitated with FLAG antibody and the immunoprecipitated complexes were subjected to *in vitro* kinase assay in the presence or absence of His-Plk1 TD or His-Plk1 KD with GST- RNA Pol II CTD as the substrate. (D) pCMV FLAG-cyclin T1 transfected 293T cells were treated with or without BI2536(1µM) for 3.5 h before harvest. The FLAG-cyclin T1 complexes were immunoprecipated with FLAG antibody and subjected to *in vitro* kinase assay with GST-RNA Pol II CTD as the substrate. Equal amount of the immunoprecipated FLAG-cyclin T1 and Cdk9 was shown. (E) FLAG-tagged cyclin T1, cyclin T1 S564A, cyclin T1 S564D over-expressed in 293T cells were immunoprecipitated with FLAG antibody and incubated with GST- RNA Pol II CTD and [γ-^32^P] ATP for the *in vitro* kinase assay. The immunoprecipated FLAG-cyclin T1 and Cdk9 were subjected to immunoblotting.

Cellular transcription oscillates greatly in a cell cycle-dependent manner. It is well known that the transcription is silenced in mitosis. The inactivation of the transcription machinery has been reported to play an important role in the repression [6–8]. However, less is known about the activity of transcription elongation complex (P-TEFb) in mitosis. To examine the P-TEFb activity in mitosis, HCT116 cells were transfected with pCMV FLAG-cyclinT1 and then synchronized in G1, S and M phases as described in material and method. The synchronization of the cells was confirmed by flow cytometry (Figure 4A, right panel). Cell lysates were harvested in the presence of phosphatase inhibitor to inhibit potential dephosphorylation and immunoprecipated with FLAG antibody. The immunoprecipated FLAG-cyclinT1 complexes were used for *in vitro* kinase assay, with purified GST-RNA Pol II CTD as the substrate. As shown in Figure 4A (left panel), P-TEFb kinase activity in mitotic cells is much lower than that in asynchronized, G1 or S phase cells. To assure if the reduction of P-TEFb activity in mitosis is caused by phosphorylation by mitotic kinases, 293T cells were transfected with pCMV FLAG-cyclin T1 and the cell lysates were immunoprecipated with FLAG antibody. The immunoprecipitated cyclin T1 complexes were first incubated as substrates with asynchronized or mitotic HeLa cell lysates for *in vitro* kinase assay. Then the supernatants were washed away, and the precipitated FLAG-cyclin T1 beads were used for the second round of *in vitro* kinase assay, with GST-RNA Pol II CTD as the substrate. Similar to the results in Figure 4A, FLAG-cyclin T1 complexes preincubated with mitotic HeLa cell lysate display decreased kinase activity towards RNA Pol II CTD (Figure 4B). Since we observed that Plk1 can phosphorylate cyclin T1 (Figure 3), we wonder if Plk1 would affect P-TEFb activity by phosphorylating cyclin T1. To address this question, 293T cells were transfected with pCMV FLAG-cyclin T1 or pCMV FLAG-Cdk9 respectively. Then FLAG-cyclin T1 or FLAG-Cdk9 were immunoprecipated with FLAG antibody, and subjected to *in vitro* kinase assay using GST-RNA Pol II CTD as the substrate, in the absence or presence of bacteria-purified His-Plk1 TD or His-Plk1 KD. The data indicated that GST-RNA Pol II CTD phosphorylation by P-TEFb from either FLAG-Cdk9 or FLAG-cyclin T1 immunoprecipitates is greatly reduced when Plk1 TD is present but not affected by Plk1 KD, and the inhibition of P-TEFb kinase activity by Plk1 TD is dose-dependent (Figure 4C). To further assure the inhibition is caused by Plk1, 293T cells were transfected with pCMV FLAG-cyclin T1 and then treated with or without Plk1 inhibitor BI2536 (1µM) for 3.5 hr before harvest. FLAG-cyclin T1 complexes were immunoprecipated and subjected to *in vitro* kinase assay with RNA Pol II CTD as the substrate. The data showed that the kinase activity of P-TEFb is much higher in the BI2536 treated sample than that in the control (Figure 4D). To further examine whether phosphorylation of cyclin T1 affects the activity of P-TEFb, 293T cells were transfected with pCMV FLAG-cyclin T1 wild-type, S564A or S564D respectively. The cell lysates were immunoprecipated with FLAG antibody and subjected to *in vitro* kinase assay with RNA Pol II CTD as the substrate. As shown in Figure 4E, the cyclin T1 S564A possesses higher kinase activity compared to the wild type cyclin T1 and the phosphomimetic mutant cyclinT1 S564D. Taken together, we propose that phosphorylation of cyclin T1 by Plk1 inhibits the P-TEFb kinase activity.

### Plk1 inhibits the P-TEFb dependent-RNA Pol II promoter transcription

**Figure 5:**
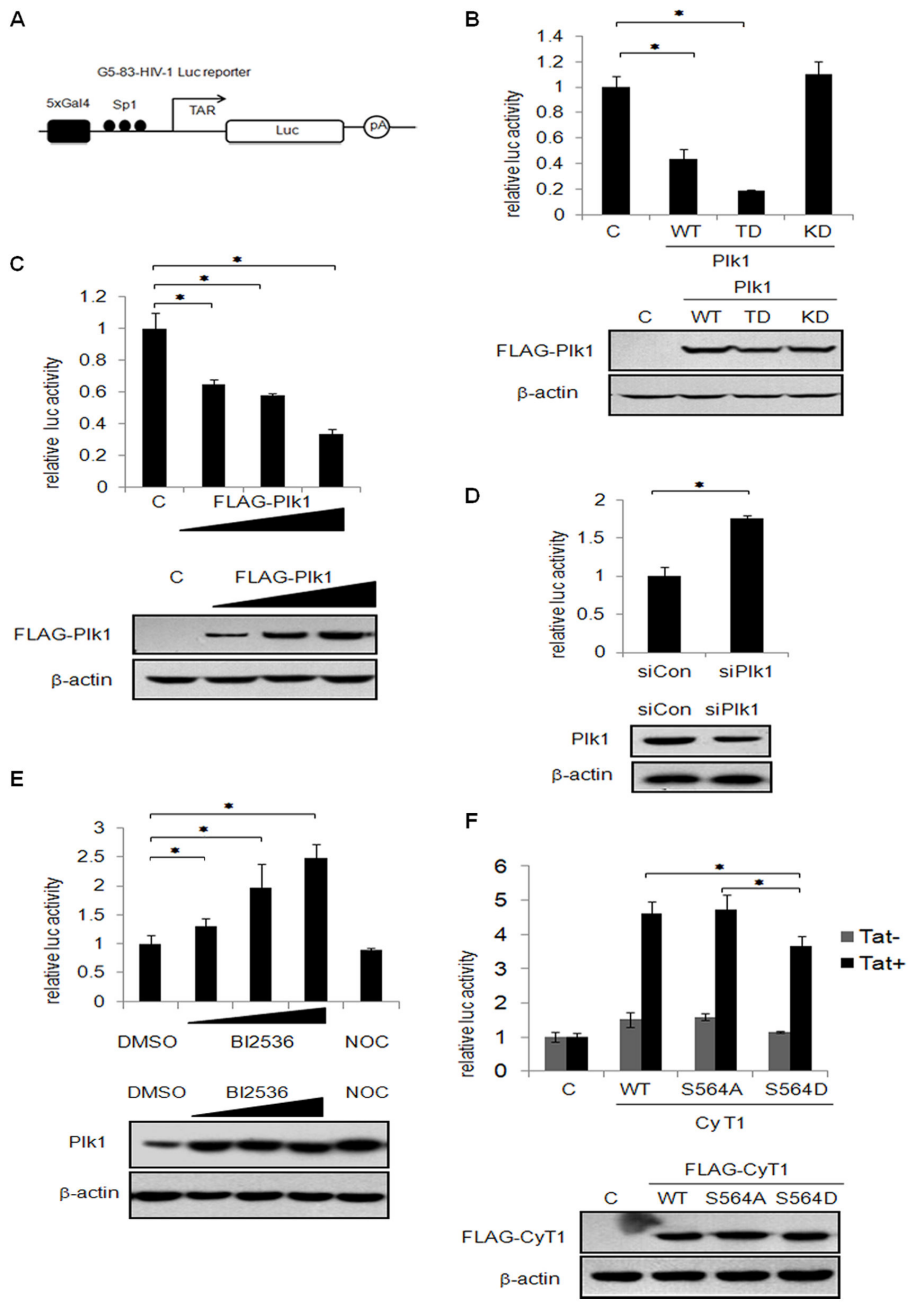
Plk1 represses P-TEFb-dependent transcription. (A) Schematic map of G5-83-HIV-Luc reporter. (B) 293T cells were co-transfected with Plk1, Plk1 TD or Plk1 KD expression plasmids and HIV-1 LTR luciferase reporter for 36 h. The cell lysates were harvested for luciferase assay. The expression level of Plk1 and its mutants was monitored by immunoblotting with FLAG antibody with β-actin as the internal control. (C) 293T cells were transfected with different doses of pCMV FLAG-Plk1 (150ng, 300ng and 600ng) and empty vector to keep an equal amount of co-tranfected DNA in each group, and with G5-83-HIV-luc luciferase reporter. After 36 h, the cell lysates were subjected to luciferase assay. The expression level of Plk1 was monitored by immunoblotting with FLAG antibody with β-actin as the internal control. (D) 293T cells were transfected with Plk1-specific siRNA or control siRNA followed by transfection with G5-83-HIV-luc luciferase reporter. The luciferase assay was performed 24 h after transfection. Immunoblotting was performed to detect the expression level of Plk1 and β-actin. (E) 293T cells were transfected with HIV-1 LTR luciferase reporter for 24 h and then treated with BI2536(100nM,500nM,1µM), Nocodazole or DMSO for 16hr before harvest. The cell lysates were subjected to luciferase assay and immunoblotting with Plk1 and β-actin. (F) NIH3T3 cells were transfected with human cyclin T1, cyclin T1 S564A, or cyclin T1 S564D expression plasmids with HIV-1 LTR reporter in the presence or absence of pCMV Tat for 24 h. The cell lysates were subjected to luciferase assay and immunoblotting with FLAG antibody and β-actin. The luciferase activity was normalized to the amount of luciferase DNA in transfected cells which was quantified by real-time PCR. The data are shown as the mean ± SD from three independent experiments. Statistical significance was determined by Students t-test (* *p value* < 0.05). “C” indicates that empty vector was used as the negative control.

The transcriptional activity of the HIV-1 long terminal repeat (LTR) is uniquely dependent on P-TEFb since it is recruited to RNA Pol II by Tat-TAR RNA to produce full-length viral transcripts [29]. To test the effect of Plk1 phosphorylation on cyclin T1 on the basal activity of the HIV-1 LTR in the absence of Tat, Plk1, Plk1 TD or Plk1 KD expression plasmids were co-transfected with an HIV-1 LTR luciferase reporter (Figure 5A). The cell lysates were then harvested for luciferase assay. As shown in Figure 5B, both wild-type Plk1 and constitutive active Plk1 (Plk1 TD) greatly inhibits the transcriptional activity of HIV-1 LTR reporter, but kinase defective Plk1 (Plk1 KD) showed no inhibition. It is worth noting that Plk1 TD displays elicited stronger inhibitory effect than wild type. Also Plk1 inhibits the HIV-1 LTR activity in a dose-dependent manner (Figure 5C). Then we took the approach of RNA interference to determine whether knockdown of Plk1 affects the transcriptional activity of HIV-1 LTR. As shown in Figure 5D, knockdown of Plk1 causes the increase of HIV-1 LTR reporter activity. We then investigated the effect of Plk1 on HIV-1 LTR reporter using Plk1 inhibitor BI2536. We transfected the cells with HIV-1 LTR reporter and then treated the cells with either Plk1 inhibitor BI2536 or Nocodazole as the control since both of them can arrest cells in prometaphase [37,38]. As shown in Figure 5E, BI2536 treatment leads to an increased activation of HIV-1 LTR reporter in a dose-dependent manner but not Nocodazole. Since murine cyclin T1 cannot interact with Tat efficiently, murine cells do not support HIV-1 transcription [29,39]. Therefore, we transfected human cyclin T1 WT, S564A or S564D expression plasmids and the HIV-1 LTR reporter with or without Tat expression plasmids into murine cells to examine the effect of cyclin T1 on the HIV-1 LTR reporter. As shown in Figure 5F, in the presence of Tat, human cyclin T1 WT and cyclin T1 S564A mutant cause about 4.5 fold increases on Tat-mediated transactivation of HIV-1 LTR reporter, while phosphomimetic mutant cyclin T1 S564D has less efficiency. Taken together, we concluded that Plk1 phosphorylates cyclin T1 at S564 and represses P-TEFb-dependent gene transcription.

### Plk1 inhibits the pseudotyped HIV-1 virus gene expression

**Figure 6:**
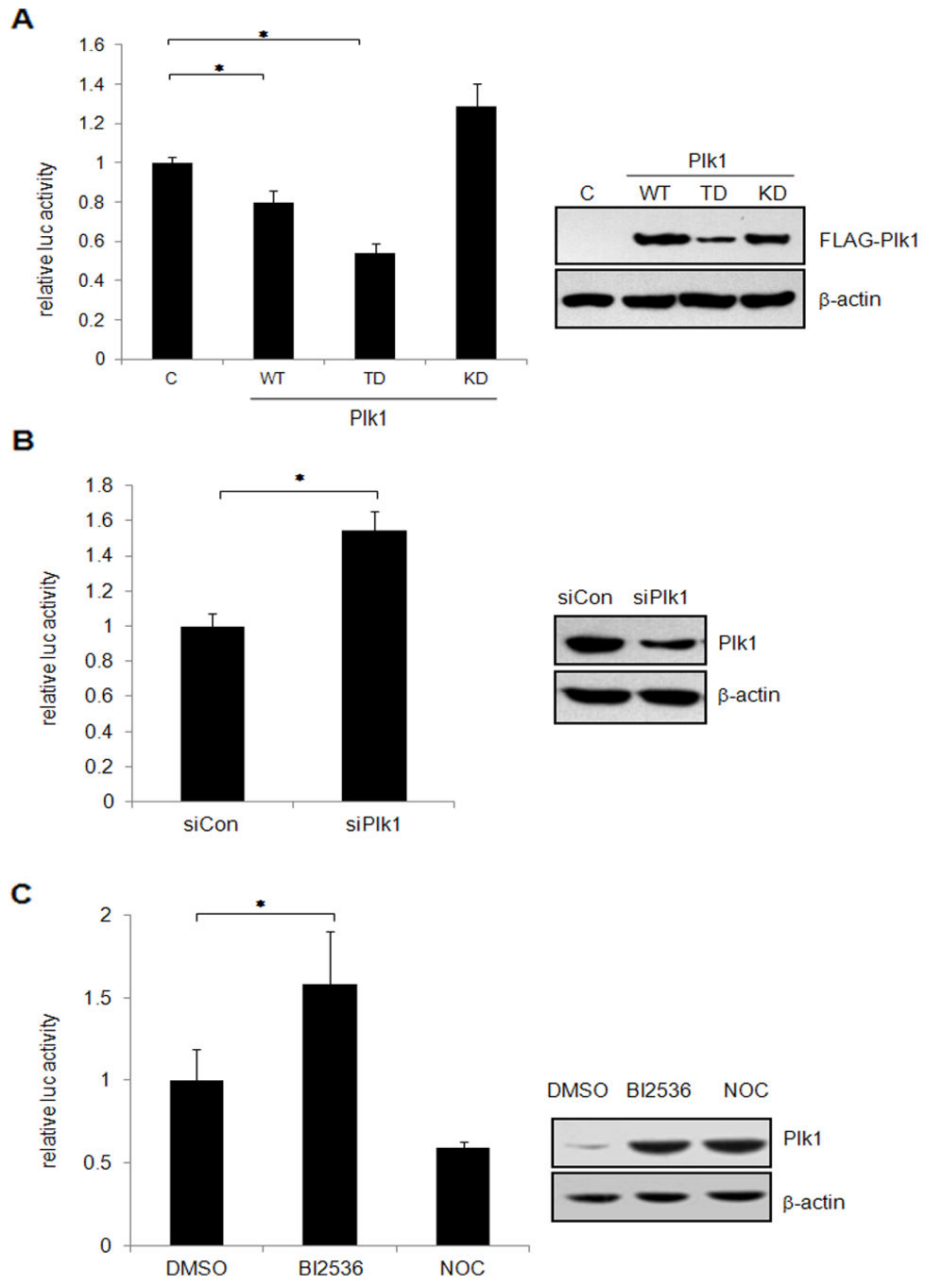
Plk1 inhibits the HIV-1 pseudovirus replication. (A) HCT116 cells were transfected with Plk1, Plk1 TD or Plk1 KD expression plasmids for 24 h and then infected with VSV-G pseudotyped pNL4.3-Luc virus for 24 h. The cell lysates were subjected to luciferase assay (left) and immunoblotting (right). “C” indicates that empty vector was used as the negative control. (B) 293T cells were transfected with Plk1-specific siRNA or control siRNA for 36 h. Then the cells were infected with VSV-G pseudotyped pNL4.3-Luc virus for 24 h. The cell lysates were harvested for luciferase assay (left) and immunoblotting (right). (C) HCT116 cells were infected with VSV-G pseudotyped pNL4.3-Luc virus for 24 h, then treated with BI2536, Nocodazole or DMSO for 16 h. The cell lysates were harvested for luciferase assay (left) and immunoblotting (right). The data are shown as the mean±SD from three independent experiments. Statistical significance was determined by Students t-test (*p value <0.05).

In order to determine the effect of Plk1 on the pseudotyped HIV-1 virus gene expression, HCT116 cells were transfected with pCMV FLAG-Plk1, pCMV FLAG-Plk1 TD or pCMV FLAG-Plk1 KD, then infected with VSV-G pseudotyped HIV-1 reporter virus. The cells lysates were harvested and subjected to luciferase assay. Consistent with the results shown in Figure 5B, both Plk1 and Plk1 TD cause the reduction on luciferase activity of the pseudotyped HIV-1 reporter significantly, but Plk1 KD has no inhibitory effect (Figure 6A). Furthermore, we performed similar experiments in 293T cells in which Plk1 was knocked down by siRNA and then infected with VSV-G pseudotyped HIV-1 reporter virus. The luciferase assay showed that knockdown of Plk1 enhances the P-TEFb-dependent gene expression of HIV-1 pseudovirus (Figure 6B). To examine if the activity of Plk1 influences the gene expression of HIV-1 pseudovirus, HCT116 cells were infected with VSV-G pseudotyped HIV-1 reporter virus and then treated with Plk1 inhibitor BI2536 or Nocodazole as the control. Consistent with the data observed in Figure 5E, luciferase assay data showed that BI2536 treatment increases the expression of HIV-1 pseudovirus reporter gene significantly although the cells were arrested in M phase, while Nocodazole treatment inhibits the gene transcription of HIV-1 pseudovirus reporter (Figure 6C). The above data indicated that inhibition of Plk1 activity favors the transcription of HIV-1 luciferase reporter. In conclusion, Plk1 negatively regulates P-TEFb-dependent gene transcription.

## Discussion

There have been several clues for polo-like kinases to regulate the cellular gene transcription. In mammals, the polo-like kinase family has 4 members, Plk1, Plk2, Plk3 and Plk4. It has been reported that polo-like kinases can phosphorylate different transcription factors and regulate their functions. For instance, Plk1 can phosphorylate FoxM1 to promote its transcriptional activity in G2/M phase [40]. Plk1 binds and phosphorylates p53, and inhibits its function as the transcriptional activator [41]. YY1 was identified to be phosphorylated by Plk1 at G2/M transition although the exact function of the phosphoryation has not been defined [36]. The phosphorylation of RNA polymerase II CTD at Thr4 by Plk3 is required for transcriptional elongation [42]. Recently, Plk1 was discovered to be involved in the direct regulation of RNA Pol III-dependent transcription in a precisely controlled manner. It promotes tRNA and 5S rRNA transcription by phosphorylating Brf1 at Ser450 (the subunit of TFIIIB) during interphase. However, as Plk1 activity peaks in mitosis, it phosphorylates Brf1 at Thr270, which prevents RNA Pol III recruitment and thus causes transcription suppression [43].

In this study, we demonstrated that as the subunits of positive RNA Pol II-dependent transcription elongation factor b (P-TEFb), Cdk9 and cyclin T1 could interact with Plk1 *in vitro* and in vivo. Plk1 binds to the histidine-rich region of cyclin T1 which is responsible for its binding with RNA Pol II C-terminal domain (CTD) [24]. It was reported that proteins interacting with cyclin T1 in the histidine-rich region play a regulatory role in P-TEFb activity. For example, the RNA Pol II CTD analogs and PIE-1 could bind to the histidine-rich region of cyclin T1 and inhibited the transcriptional elongation [30]. The growth factor Granulin interacted with cyclin T1 in the histidine-rich region and repressed the transcriptional activity of HIV-1 promoter [31]. These results suggested that Plk1, as a cyclin T1 histidine-rich region interacting protein could also modulate P-TEFb activity.

Posttranslational modifications of P-TEFb play important roles in P-TEFb-dependent transcription regulation. Acetylation of Cdk9 increases the P-TEFb elongation activity [25], while cyclin T1 acetylation by p300 triggers the dissociation of HEXIM 1 and 7SK snRNA from cyclin T1/Cdk9 and activates P-TEFb [44]. Autophosphorylation of Cdk9 appears to regulate P-TEFb transcription elongation activity dynamically [45]. Human cyclin T1 was originally identified as a cellular phosphoprotein that associates specifically with the transactivation domain of the HIV-1 Tat protein [32]. It can be phosphorylated by Cdk9 at its C-terminal region although the precise phosphorylation sites and the biological significance for its phosphorylation remain unknown [34]. In this study, we demonstrated that cyclin T1 is a substrate of Plk1. The phosphorylation sites of cyclin T1 are located at its C-terminal region. Mass spectrometry analysis showed that cyclin T1 Ser564 is one of the phosphorylation sites, while the data from *in vitro* kinase assay using cyclin T1 S564A mutant indicated that there are still other phosphorylation site(s). The phenomenon that cyclin T1 Ser564 mutant does not abolish the P-TEFb activity totally (Figures 4E and 5F) could be due to the abundance of endogenous wild type cyclin T1 or the need for other modifications on cyclin T1. The precise other phosphorylation sites on cyclin T1 by Plk1 or other kinase *in vivo* and the functions still remain to be clarified.

P-TEFb activity regulated by Plk1-dependent phosphorylation seems reasonable since the transcription is silenced due to the phosphorylation of components involved in transcription in mitosis [6–8] and the activity of Plk1 peaks in mitosis. However, there may be other mitotic components that could inhibit P-TEFb kinase activity in addition to Plk1 or other kinases up-regulating P-TEFb activity in interphase but losing their kinase activity in mitosis. Brd4 can be such a candidate. Brd4 was recently identified as an atypical kinase that can phosphorylate CTD of RNA Pol II [46], in addition to its traditional function to recruit P-TEFb to chromatin [21,22]. Since Brd4 could associate with cyclin T1 directly, it may also regulate P-TEFb activity by phosphorylating cyclin T1. Interestingly, Brd4 can be phosphorylated by Plk1 *in vitro* [47]. So, how Brd4, P-TEFb and Plk1 cross-talk with each other and regulate transcription are yet to be determined.
